# Comparative diagnostic performance of ACR 1997, SLICC 2012, and EULAR/ACR 2019 criteria in childhood-onset systemic lupus erythematosus: A retrospective study from a tertiary care centre in south India

**DOI:** 10.1515/rir-2026-0006

**Published:** 2026-03-30

**Authors:** Sabarinath Mahadevan, Balakrishnan Navaneethakrishnan, Mahabaleshwar Mamadapur, Harshwardhan Patil

**Affiliations:** Institute of Rheumatology, Madras Medical College, Chennai, Tamilnadu, India; Department of Clinical Immunology and Rheumatology, JSS Medical College and Hospital, JSS Academy of Higher Research, Mysore 570004, Karnataka, India; Department of Pharmacy Practice, JSS College of Pharmacy, JSS Academy of Higher Research, Mysuru 570004, Karnataka, India

**Keywords:** systemic lupus erythematosus, childhood-onset systemic lupus erythematosus, classification criteria, EULAR/ACR 2019, SLICC 2012, ACR 1997, sensitivity

## Abstract

**Background and Objective:**

Childhood-onset systemic lupus erythematosus (cSLE) accounts for 10%–20% of overall lupus cases. Data on the epidemiology of cSLE in our population are limited, and there are no exclusive classification criteria tailored for childhood lupus. The primary objective was to describe the epidemiological and clinical profile of cSLE in our centre. The secondary objective was to evaluate the performance of the 1997 American College of Rheumatology (ACR), the 2012 Systemic Lupus International Collaborating Clinics (SLICC), and the European Alliance of Associations for Rheumatology/ American College of Rheumatology (EULAR/ACR) 2019 classification criteria in cSLE.

**Methods:**

We conducted a retrospective medical record review of children with a clinical diagnosis of systemic lupus erythematosus (SLE) at our tertiary care centre between January 2024 and July 2024. Children with overlap connective tissue diseases were excluded. All three classification criteria were applied and compared against the gold standard of physician diagnosis. Clinical and laboratory features were analysed, and the sensitivity of each set of criteria was calculated.

**Results:**

Sixty-six children with clinically confirmed cSLE were included. The female-to-male ratio was 5.6: 1, with a median age at symptom onset of 15.5 years and a median diagnostic delay of 6 months. The most frequent manifestations were fever (83.0%), alopecia (70.0%), arthritis (64.0%), oral ulcers (39.0%), acute cutaneous lupus (67.0%), leukopenia (24.0%), and biopsy-proven lupus nephritis (31.0%). The SLICC 2012 criteria demonstrated the highest sensitivity (95.5%), followed by EULAR/ACR 2019 (93.9%) and ACR 1997 (84.8%). Notably, 4.5% of clinically diagnosed cases did not fulfil any existing criteria. Alopecia, hypocomplementemia, and biopsy-proven nephritis were frequently missed by ACR 1997 but captured by SLICC 2012.

**Conclusion:**

The SLICC 2012 criteria showed superior sensitivity compared to ACR 1997, while EULAR/ACR 2019 also performed well. However, a small subset of clinically diagnosed cSLE cases remained unclassified by all criteria, highlighting the need for pediatric-specific classification frameworks.

## Introduction

Systemic lupus erythematosus (SLE) is a chronic, multisystem autoimmune disorder characterized by heterogeneous clinical manifestations and the presence of diverse autoantibodies. Approximately 10%–20% of SLE cases manifest within the age group of 16 years, a form known as childhood-onset SLE (cSLE).^[1, 2, 3, 4, 5, 6]^ Compared to adult-onset SLE, cSLE is typically associated with a more aggressive disease course, increased disease activity, and a higher risk of major organ involvement, particularly in the renal, neurologic, and hematologic systems. This leads to greater cumulative damage, morbidity, and mortality in affected children and adolescents.^[7,8]^

The diagnosis of SLE, irrespective of age at onset, is inherently challenging due to the broad spectrum of clinical presentations and overlap with other autoimmune, infectious, or hematologic disorders. Notably, previous studies indicate that the clinical presentation of childhood-onset SLE varies across ethnicities, with Asian cohorts such as ours frequently demonstrating higher rates of renal involvement compared to Western populations. To facilitate consistent identification of SLE for research and epidemiological purposes, various classification criteria have been developed.^[9]^ Historically, the 1982 American College of Rheumatology (ACR) criteria and its 1997 revision were widely adopted but were criticized for limited sensitivity, especially in early or atypical presentations.

The Systemic Lupus International Collaborating Clinics (SLICC) classification criteria, introduced in 2012,^[8,10,11,12,13]^ were designed to address several limitations of the 1997 ACR criteria. Compared to the ACR criteria, the SLICC system demonstrated higher sensitivity (92%–99% *vs*. 77%–91%) but at the cost of reduced specificity (74%–88% *vs*. 91%–96%). Nevertheless, both the ACR and SLICC frameworks rely on a simple count of fulfilled criteria, without accounting for disease severity, weighting of features, or clinical heterogeneity.^[12, 13, 14]^

To overcome some of these limitations, a novel classification system was developed jointly by the European Alliance of Associations for Rheumatology (EULAR) and the ACR in 2019.^[15]^ The EULAR-ACR 2019 criteria are the most recent internationally accepted classification system for SLE, combining weighted clinical and immunologic features to improve diagnostic accuracy, particularly in early or atypical cases. This updated set of criteria introduced antinuclear antibodies (ANA) positivity as a mandatory entry criterion and employed a weighted scoring approach for clinical and immunologic features, aiming to enhance diagnostic precision by balancing sensitivity and specificity more effectively.^[16]^

Despite recent advances in SLE classification, it is important to recognise that all major criteria—ACR 1997, SLICC 2012, and EULAR/ACR 2019 have been primarily developed and validated in adult populations. Their utility in pediatric cohorts remains insufficiently explored, despite the fact that cSLE is typically characterised by higher disease activity, distinct clinical manifestations, and greater severity of organ involvement compared to adult-onset disease. The lack of pediatric-specific classification criteria raises concerns regarding the accuracy and timeliness of diagnosis when adult-based frameworks are applied to children and adolescents.^[17, 18, 19, 20, 21, 22]^

Moreover, ethnicity has been shown to significantly influence SLE susceptibility, clinical phenotype, and disease course, underscoring the importance of evaluating classification criteria in ethnically diverse populations. In this context, the present study aims to compare the diagnostic performance of the ACR 1997, SLICC 2012, and EULAR/ACR 2019 classification criteria in identifying children with SLE in a South Indian cohort from a tertiary care center. The performance of each set of criteria was assessed. This study seeks to evaluate their sensitivity, applicability in pediatric clinical practice, and ability to identify cSLE cases that may be missed by earlier criteria, while also providing insight into the potential need for region- and age-specific diagnostic frameworks.

## Materials and Methods

### Study Design and Setting

This was a retrospective observational medical record review study conducted at a tertiary care referral centre in South India. The study involved a detailed review of medical records from patients attending the dedicated lupus clinic between January 2024 and July 2024.

### Study Population

Children diagnosed with SLE before the age of 18 years attending the outpatient and inpatient departments were included in the study. All patients were either diagnosed and followed at our centre or were referred from outside centres with a suspected diagnosis of SLE, subsequently confirmed and managed at our institution by a qualified rheumatologist.

### Inclusion Criteria

Children were eligible for inclusion if the first onset of symptoms suggestive of SLE occurred before 16 years of age, in accordance with standard definitions distinguishing childhood-onset from adult-onset disease. The age at initial symptom onset, rather than the age at diagnosis, was used for classification purposes. Exclusion Criteria.

Patients with overlap syndromes or coexisting connective tissue diseases, including juvenile dermatomyositis and mixed connective tissue disease, were excluded from the study. A total of 70 patients met the inclusion criteria. After exclusion of 4 patients with overlap syndromes, 66 children with cSLE were included in the final analysis.

### Data Collection

Clinical and laboratory data at the time of the first presentation were retrieved from medical records. The following were documented:

- Demographic details: age at symptom onset, gender.
- Clinical features and organ involvement at baseline.
- Laboratory findings relevant to the ACR 1997, SLICC 2012, and EULAR/ACR 2019 criteria.

The ACR 1997, SLICC 2012, and EULAR/ACR 2019 criteria sets were applied for classification purposes only. As these criteria are not designed for diagnosis, the clinical diagnosis by pediatric rheumatologists served as the reference standard for comparison. Definitions of each domain were applied as per the original validation criteria for ACR 1997, SLICC 2012, and EULAR/ACR 2019. ANA were tested using indirect immunofluorescence (≥ 1: 80 titer as positive). Anti-double-stranded DNA (anti-dsDNA) antibodies were measured using enzyme-linked immunosorbent assay (ELISA). Anti-cardiolipin IgG and IgM (ACL) antibodies were done in all patients. Lupus anticoagulant and anti-beta2-glycoprotein I antibodies were tested only in selected patients with clinical suspicion of antiphospholipid syndrome and negative ACL due to resource limitations. Renal biopsy was performed in patients with urinary abnormalities suggestive of lupus nephritis.

### Classification Criteria Application

Each Patient’s Data Was Evaluated for the Presence of Classification Domains Under:

- ACR 1997 (≥ 4 of 11 criteria),
- SLICC 2012 (≥4 criteria, including at least one clinical and one immunological, or biopsy-proven lupus nephritis with ANA/anti-dsDNA), and
- EULAR/ACR 2019 (ANA positivity as entry criterion, total weighted score ≥10).

Patients were categorized into mutually exclusive groups based on fulfillment of the different classification criteria. Group 1 included patients who satisfied both the ACR 1997 and SLICC 2012 classification criteria. Group 2 comprised patients who fulfilled the SLICC 2012 criteria but did not meet the ACR 1997 criteria. Group 3 consisted of patients who met the ACR 1997 criteria but failed to fulfill the SLICC 2012 criteria. Group 4 included patients who did not meet either the ACR 1997 or SLICC 2012 criteria but were clinically diagnosed with SLE based on expert consensus among three pediatric rheumatologists, supported by clinical judgment and longitudinal follow-up. Group 5 comprised patients who fulfilled the EULAR/ACR 2019 classification criteria but did not meet the SLICC 2012 criteria. Group 6 included patients who satisfied all three classification criteria, namely ACR 1997, SLICC 2012, and EULAR/ACR 2019. Group 7 consisted of patients who fulfilled only the SLICC 2012 classification criteria without meeting either the ACR 1997 or EULAR/ACR 2019 criteria. Group 8 included patients who satisfied both the SLICC 2012 and EULAR/ACR 2019 criteria but did not meet the ACR 1997 criteria.

### Statistical Analysis

For statistical analysis, baseline demographic and clinical data were summarized using descriptive statistics, with numerical variables presented as mean ± standard deviation or median (interquartile range) based on their distribution, and categorical variables as frequencies and percentages. The sensitivity of each classification criteria set (ACR 1997, SLICC 2012, EULAR/ACR 2019) was calculated. McNemar’s test was utilized to compare the paired proportions of patients satisfying the different classification criteria. The computed Cohen’s κ statistic was used for each criterion pairing to quantify agreement beyond chance (interpretation: poor to moderate). A *P*-value of < 0.05 was considered statistically significant, and κ values were used to assess concordance in case capture.

All analyses were performed using IBM SPSS Statistics for Windows, Version 20.0.

### Ethical Considerations

The study protocol was approved by the Institutional Ethics Committee (IEC). Given its retrospective design, the requirement for patient consent was waived.

## Results

A total of 66 pediatric patients with a confirmed clinical diagnosis of cSLE were included in the analysis. Females constituted 84% (*n* = 56) of the cohort. All participants were residents of southern India and belonged predominantly to the Dravidian ethnic group, reflecting the regional demographic profile of our tertiary care center. The female-to-male ratio was 5.6:1. The median age at symptom onset was 15.5 years. The median interval from symptom onset to diagnosis was 6 months. Arthritis was seen in 64% (42/66) of cases, fever in 83% (55/66), leukopenia in 24% (16/66), and nephritis in 38% (25/66) (Table 1). Notably, 7 patients (11%) experienced diagnostic delays exceeding 12 months. Presenting features in these delayed cases included arthritis (*n* = 2), immune thrombocytopenia (*n* = 2), cutaneous lesions (*n* = 2), and abdominal vasculitis (*n* = 1). In our cohort, the most common clinical domains positive in ACR, SLICC, and EULAR/ ACR were fever, alopecia and arthritis. The most common immunological domain positive in all three criteria was ANA positivity (*n* = 62, 94%).

**Table 1:** Clinical and immunological characteristics of the SLE cohort (n = 66)

Domain	Criteria	*n* = 66
Clinical Domai		
Constitutional	Fever	55 (83%)
Mucocutaneous manifestations	Nonscarring alopecia	46 (69.69%)
	Oral ulcers	26 (39.39%)
	Subacute cutaneous or discoid lupus	9 (13.63%)
	Acute cutaneous lupus	29 (66.66%)
Arthritis	> 2 joints with synovitis, or > 2 tender joints with morning stiffness > 30 min	42 (63.63%)
Neurologic	Delirium Psychosis Seizure	10 (15.15%)
Serositis	Pleural or pericardial effusion	13 (19.69%)
	Acute pericarditis	0 (0%)
Hematologic	Leukopenia	16 (24.24%)
	Thrombocytopenia	16 (24.24%)
	Autoimmune hemolytic anemia	18 (27.27%)
Renal	Proteinuria > 0.5 g/24 h	25 (37.87%)
	Class II or V lupus nephritis	5 (7.57%)
	Class III or IV lupus nephritis	15 (22.72%)
Immunologic Domain		
ANA	ANA positive, titer ≥ 1: 80	62 (94%)
Complement protein	Low C3 or low C4	9 (13.63%)
	Low C3 and low C4	28 (42.42%
Highly specific antibodies domain	Anti-dsDNA antibody	50 (75.75%)
	Anti-Smith antibody	26 (39.39%)

Mucocutaneous manifestations include acute/subacute cutaneous lupus, discoid lesions, oral ulcers, and nonscarring alopecia (as per EULAR/ACR 2019 criteria). ACR: American College of Rheumatology.

### Comparative Diagnostic Performance of Classification Criteria

The diagnostic accuracy of the ACR 1997, SLICC 2012, and EULAR/ACR 2019 criteria was assessed using sensitivity. The detailed metrics are presented in Table 2.

**Table 2:** Performance measures for ACR 1997, SLICC 2012, and EULAR/ACR 2019 criteria

Classification set	Number of patients meeting criteria	Sensitivity (%)	95% CI
ACR 1997	56/66	84.8	76.2 – 93.5
SLICC 2012	63/66	95.5	90.4 – 100.0
EULAR/ACR 2019	62/66	93.9	88.2 – 99.7

ACR, American College of Rheumatology; SLICC, Systemic Lupus International Collaborating Clinics.

In our study, 85% (*n* = 56 patients) satisfied > 4 ACR criteria compared to 96% (*n* = 63) of patients who satisfied > 4 SLICC criteria and 94% (*n* = 62) who satisfied the ACR/EULAR 2019 criteria. The higher sensitivity observed with the SLICC 2012 and EULAR/ACR 2019 criteria compared to the ACR 1997 criteria was statistically significant (*P* = 0.015). The mean scores of ACR, SLICC, and ACR 2019 criteria in our cohort were 5.25, 6.75, and 23.29, respectively.

The cohort distribution across the six observed groups was as follows: Group 1: 84% (*n* = 56) of patients satisfied both ACR 1997 and SLICC 2012 criteria. Group 2: 10% (*n* = 7) of patients satisfied SLICC 2012 but not ACR 1997 criteria.

Group 3: None of the patients who met ACR 1997 failed to meet SLICC 2012 criteria. Group 4: 4.5% (*n* = 3) of patients did not meet either ACR 1997 or SLICC 2012 criteria but were classified as SLE based on expert consensus. Their SLICC scores were 3, while ACR scores were 2 (*n* = 2) and 1 (*n* = 1), underscoring the limitations of rigid criteria in recognizing incomplete or evolving pediatric disease. Group 5: No patients fulfilled EULAR/ACR 2019 criteria without meeting SLICC 2012. Group 6: 55 patients met ACR 1997, SLICC 2012 and ACR/EULAR 2019 criteria. Group 7: No Patients Fulfilled SLICC 2012 Alone without Meeting Either ACR 1997 or EULAR/ACR 2019. Group 8: 63 patients fulfilled both the SLICC 2012 and EULAR/ACR 2019 criteria.

### Patients with Discordant Classification across Criteria

During comparison, seven patients who were ACR-negative met the SLICC criteria. Six had an ACR score of 3, and one had a score of 2 but met the SLICC 2012 criteria through biopsy-proven lupus nephritis. The specific SLICC components contributing to classification in this subgroup are summarised in Table 3.

**Table 3:** SLICC-specific components are positive in ACR-negative lupus patients

S. No	SLICC-specific components not included in the ACR criteria	Patients with ACR score of 3 (*n* = 6)	Patients with ACR score of 2 (*n* = 1)
1.	Hypocomplementemia	3	
2.	Alopecia	3	
3.	Positive direct coomb’s test without hemolysis	1	
4.	Separate Hematological components-Anemia, thrombocytopenia	2	
5.	CNS involvement	1	
6.	Biopsy-proven lupus nephritis		1

SLICC, systemic lupus international collaborating clinics.

One patient with alopecia, serositis, and anti-dsDNA positivity (total SLICC score = 3) nevertheless fulfilled the ACR/EULAR 2019 criteria due to the additional presence of fever, resulting in a weighted score of ≥ 10.

### Summary of Diagnostic Implications

These findings collectively demonstrate that SLICC 2012 improves sensitivity and domain inclusivity over ACR 1997, while EULAR/ACR 2019 also offers optimal sensitivity in this pediatric cohort (Table 3 and 4).

**Table 4:** McNemar’s test results

Comparison	b	c	McNemar *P*-value
SLICC *vs*. ACR 1997	7	0	0.0156
EULAR *vs*. ACR 1997	7	0	0.0156
SLICC *vs*. EULAR	3	1	0.6250

b = Criterion A positive, Criterion B negative; c = Criterion A negative, Criterion B positive.

SLICC 2012 demonstrates the highest sensitivity (95.5%), significantly outperforming ACR 1997 (84.8%) (*P* = 0.0156). ACR/EULAR 2019 also significantly exceeds ACR 1997 (93.9% *vs*. 84.8%, *P* = 0.0156). There is no significant difference between SLICC 2012 and ACR/EULAR 2019 (*P* = 0.625). Agreement metrics reflect that newer criteria overlap substantially but identify some distinct cases. To place our findings in a broader context, we compared the sensitivity of the three classification criteria observed in our cohort with results reported from other pediatric SLE studies worldwide.

As summarized in Table 5, the sensitivity of the SLICC 2012 and EULAR/ACR 2019 criteria consistently exceeded that of the ACR 1997 criteria across multiple international cohorts. The performance trends in the present study (SLICC 96%, EULAR/ACR 94%, ACR 85%) closely mirror those reported from Israel, Brazil, Oman, and Turkey, reinforcing the superior sensitivity of the newer criteria in childhood-onset SLE populations.

**Table 5:** Comparison of sensitivity of ACR 1997, SLICC 2012, and EULAR/ACR 2019 classification criteria in childhood-onset SLE cohorts

Study (year)	Country/region	*n* (cSLE)	Sensitivity ACR 1997	Sensitivity SLICC 2012	Sensitivity EULAR/ACR 2019
Aslan 2025^[1]^	Turkey (single-centre)	111	94%	98%	98%
Fonseca 2019^[2]^	Brazil (single-centre)	122	71%	89%	88%
Levinsky 2021^[21]^	Israel (multicentre)	112	79%	96%	96%
Abdwani 2021^[30]^	Oman (multicentre)	113	66%	86%	89%
Present study	India (single-centre)	66	85%	96%	94%

cSLE, childhood-onset systemic lupus erythematosus; ACR, American college of rheumatology; SLICC, systemic lupus international collaborating clinics.

## Discussion

This study presents a comprehensive comparative evaluation of three widely used SLE classification systems—ACR 1997, SLICC 2012, and EULAR/ACR 2019—in diagnosing cSLE within a South Indian cohort. Our definition of cSLE adhered to the internationally accepted cut-off of symptom onset before 16 years of age, ensuring comparability with other paediatric cohorts. Furthermore, acknowledging the significant geographical and ethnic variations in SLE clinical manifestations, influenced by both genetic and non-genetic factors, it is imperative to assess the performance of these criteria across diverse populations.

The demographic profile of our cohort reflects the well-recognized female predominance of cSLE, consistent with global and Indian reports. Although the age at symptom onset and diagnosis delay in our study align with prior Indian data, the persistence of diagnostic lag underscores the continuing need for early recognition in pediatric practice. Previous studies have similarly highlighted that delayed diagnosis contributes to organ damage accrual and higher disease activity at presentation.^[22]^ A study from eastern India on childhood lupus by Mondal *et al* observed a female-to-male ratio of 3.8:1.^[23]^ In a study by Careno *et al*, the female-to-male ratio was 6:1. In our cohort, the median age at the onset of illness was 15.5 years.^[24]^

It is important to emphasize that ACR 1997, SLICC 2012, and EULAR/ACR 2019 are classification criteria, not diagnostic criteria. Their primary aim is to create uniform patient cohorts for clinical research rather than to guide individual diagnosis. In this study, the physician’s expert judgment was used as the gold standard, and the sensitivity of each classification set was assessed relative to this clinical diagnosis. Misclassification in this context therefore reflects differences in criteria sensitivity rather than diagnostic error. Comparative evaluation of classification criteria remains critical, as paediatric patients often exhibit incomplete or evolving disease phenotypes. Our findings reinforce observations from other cohorts that the SLICC 2012 and EULAR/ACR 2019 criteria demonstrate greater sensitivity than the ACR 1997 set. The enhanced performance of these newer frameworks likely reflects broader inclusion of immunologic and renal parameters, which are particularly relevant in children. However, consistent with previous Indian and Asian studies, none of the criteria achieved universal applicability, highlighting the need for paediatric-specific validation

The median duration from the onset of symptoms to the time taken for diagnosis was 6 months. In a study done by Aggarwal *et al*. among children with lupus in India, the median age at onset of illness was 14 years, and the median duration of illness prior to diagnosis was 1 year.^[25]^ The average age at disease onset was 13.04 in a study by Careno *et al*. on childhood lupus till 18 years.^[24]^ In our cohort, 7 cases were diagnosed after 1 year of the onset of the initial symptom. The longer time taken for diagnosis was because of SLE presenting with predominant symptoms of arthritis (*n* = 2), immune thrombocytopenic purpura (*n* = 2), skin lesions (*n* = 2) and abdominal vasculitis (*n* = 1). A study by Sakamoto *et al*, showed that chronic arthritis was an isolated disease manifestation observed at disease onset in 41% of childhood lupus patients, masquerading as juvenile idiopathic arthritis (JIA).^[26]^

The most common clinical domain positive in our study was arthritis (in ACR), alopecia (in SLICC) and constitutional (in ACR/EULAR 2019). In the study by Mondal *et al*, the most common domains positive were renal (54%), haematological (54%) and neuropsychiatric features (25%). Joints (68%) and skin (54%) were the most common minor organ involved in their study. In a meta-analysis done by Pravesh Kumar Bundhun *et al*, 1560 childhood-onset SLE patients were analysed. They observed that haematological, renal, central nervous system (CNS), ocular, vasculitis and fever were significantly higher in childhood SLE when compared with adult SLE.^[27,28]^ In another study by Hiraki *et al*, the most common clinical manifestations were arthritis (67%), malar rash (66%), nephritis (55%), and CNS disease (27%).^[28]^

The sensitivities of ACR, SLICC and ACR/EULAR 2019 criteria in our cohort were 84%, 95% and 94% respectively. The few discordant cases in our study provide valuable insights into domains where pediatric presentations diverge from adult-derived criteria. Children often manifest hematologic or renal involvement early, sometimes before the accumulation of multiple mucocutaneous or immunologic criteria required by the ACR 1997 or EULAR/ACR 2019 criteria. These findings echo earlier pediatric series suggesting that criteria weighting toward serology and mucocutaneous features may under-recognize organ-predominant cSLE. Tailored pediatric validation studies are therefore warranted to ensure timely recognition of such cases.

Similar studies (Table 5) have been done in different populations assessing the performance of SLICC and ACR criteria in children. The higher sensitivity of SLICC remained when patients were stratified by sex. The mean SLICC and ACR scores in our study were 6.7 and 5.2, respectively. In a study done by Jessie *et al*., the mean scores of SLICC and ACR were 7.5 and 5.7, respectively.^[29]^

Batu *et al*.^[17]^ described the performance of these criteria in a large cohort of 262 cSLE patients from Turkey at diagnosis. They reported the highest sensitivity for SLICC 2012 (95.4%), followed by EULAR/ACR 2019 (91.5%), and then ACR 1997 (68.7%). In contrast, ACR 1997 showed the highest specificity (94.8%), compared to SLICC 2012 (89.7%) and EULAR/ACR 2019 (88.5%). Fonseca *et al*.^[20]^ similarly described the performance of the three classifications in a cSLE cohort of 122 patients from Brazil, at diagnosis and at one-year follow-up. They reported the highest sensitivity with SLICC 2012 at diagnosis (89.3%) and one-year follow-up (97.5%), compared to ACR 1997 (70.5% and 95.1%) and EULAR/ACR 2019 (87.7% and 95.1%). The specificity for ACR 1997 was highest at diagnosis (83.2%) and one-year follow-up (76.4%), compared to SLICC 2012 (80.9% and 76.4%) and EULAR/ACR 2019 (67.4% and 58.4%). In a multicentre study from Oman, Abdwani *et al*.^[30]^ evaluated 113 children with cSLE and reported progressively higher sensitivities for ACR 1997 (66%), SLICC 2012 (86%), and EULAR/ACR 2019 (89%).

Our study is strengthened by its real-world clinical design, rigorous diagnostic confirmation by pediatric rheumatologists, and detailed comparison with existing literature. The absence of patients in the SLICC-only or SLICC + EULAR/ ACR (without ACR 1997) groups in our cohort likely reflects the considerable clinical overlap captured by the newer criteria and the modest sample size. In pediatric-onset disease, characteristic multi-domain involvement often allows simultaneous fulfillment of multiple criteria sets. Larger multicentric datasets may help identify rare phenotypes, such as isolated neurologic or Coombs-positive presentations, that fulfill only SLICC 2012.

This study has several important limitations that should be considered when interpreting the findings. Being a single-centre, retrospective analysis, it is inherently subject to selection bias. The absence of a control group or true negative cases restricted the assessment of specificity, predictive values, and likelihood ratios—parameters essential for evaluating the complete discriminatory performance of classification criteria. Consequently, the comparative analysis focuses solely on sensitivity. The modest sample size further limited the statistical power of subgroup analyses and prevented reliable estimation of specificity, as only clinically confirmed cSLE cases were included. Therefore, the study primarily reflects the relative sensitivity of the three classification criteria rather than their absolute diagnostic accuracy. Moreover, as all participants were recruited from a single centre in southern India, the cohort lacked ethnic diversity, and the findings may not fully represent the heterogeneity of SLE manifestations across different regions or global populations.

The relatively small sample size (*n* = 66), although reflective of our referral base, results in wider confidence intervals and limited statistical power for subgroup analyses, such as nephritis-only presentations. Additionally, our analysis was confined to baseline data without evaluating longitudinal accrual of criteria; some patients may have fulfilled further items over time, influencing comparative sensitivity trends. Together, these constraints underscore the need for larger, prospective, multi-centre studies with appropriate control groups to fully validate and refine pediatric SLE classification criteria.

## Conclusion

In this retrospective study, we analysed the epidemiology and classification characteristics of childhood and adolescent-onset SLE in a South Indian cohort. Our findings demonstrate that the SLICC 2012 classification criteria exhibit higher sensitivity than the ACR 1997 criteria, identifying additional patients who would otherwise be missed. Moreover, the recently introduced EULAR/ACR 2019 criteria demonstrated perfect sensitivity, highlighting their potential utility in pediatric populations.

Importantly, our results underscore the limitations of applying adult-derived classification frameworks to children, as a small subset of clinically confirmed cSLE cases did not fulfill any formal classification criteria. This gap reinforces the need for flexible, clinically grounded approaches and the development of pediatric-specific criteria that reflect the unique disease phenotype and presentation in younger patients.
